# Evaluation of Fatigue Behavior of Asphalt Field Cores Using Discrete Element Modeling

**DOI:** 10.3390/ma17133108

**Published:** 2024-06-25

**Authors:** Min Xiao, Yu Chen, Haohao Feng, Tingting Huang, Kai Xiong, Yaoting Zhu

**Affiliations:** 1 Jiangxi Provincial Communications Investment Group Co., Ltd., Project Construction Management Company, Nanchang 330200, China; xmkkxx2024@163.com (M.X.); xiongkai9996@163.com (K.X.); 2 Hubei Highway Engineering Research Center, School of Transportation and Logistics Engineering, Wuhan University of Technology, Wuhan 430063, China; fenghaohao@whut.edu.cn (H.F.); huangtingting@whut.edu.cn (T.H.); 3 Zhejiang Communications Construction Group Co., Ltd., Design Institute Branch, Hangzhou 310051, China; 4 Jiangxi Transportation Institute Co., Ltd., Nanchang 330200, China; zhuyt7538@163.com

**Keywords:** fatigue behavior, asphalt field cores, discrete element method (DEM), semi-circular bending (SCB) test

## Abstract

Fatigue cracking is one of the primary distresses of asphalt pavements, which significantly affects the asphalt pavement performance. The fatigue behavior of the asphalt mixture observed in the laboratory test can vary depending on the type of fatigue test and the dimension and shape of the test specimen. The variations can make it difficult to accurately evaluate the fatigue properties of the field asphalt concrete. Accordingly, this study proposed a reliable method to evaluate the fatigue behavior of the asphalt field cores based on discrete element modeling (DEM). The mesoscopic geometric model was built using discrete element software PFC (Particle Flow Code) and CT scan images of the asphalt field cores. The virtual fatigue test was simulated in accordance with the semi-circular bending (SCB) test. The mesoscopic parameters of the contacting model in the virtual test were determined through the uniaxial compression dynamic modulus test and SCB test. Based on the virtual SCB test, the displacement, contact forces, and crack growth were analyzed. The test results show that the fatigue life simulated in the virtual test was consistent with that of the SCB fatigue test. The fatigue cracks in the asphalt mixture were observed in three stages, i.e., crack initiation, crack propagation, and failure. It was found that the crack propagation stage consumes a significant portion of the fatigue life since the tensile contact forces mainly increase in this stage.

## 1. Introduction

Fatigue cracking is one of the most common distresses of asphalt pavement. Crack initiation and propagation can reduce the bearing capacity of the pavement structure and allow the penetration of water into the structure, resulting in other distresses such as water damage [1]. Fatigue cracking is mainly caused by repeated traffic loading. The repeated loading can lead to the micro-cracks forming and merging, eventually propagating into macro-cracks, i.e., alligator cracking, which can weaken the overall structural capacity of the asphalt pavement and deteriorate the bonding between asphalt and aggregates [2]. Therefore, it is of significance to evaluate the fatigue behavior of the asphalt mixture, which could help prolong the fatigue life. 

Most of the existing studies have primarily analyzed the fatigue behavior of the asphalt mixture using laboratory tests and summarized the mechanism of fatigue cracking [3,4,5]. However, most of the previous studies focused on the properties of lab-fabricated asphalt mixture specimens and were limited when evaluating the properties of the core samples, which can lead to inefficient utilization of the core samples. Moreover, in practical pavement applications, asphalt pavement is constructed through three phases of compaction and ages under in-service conditions, which are subject to environmental fluctuations, such as temperature variation, precipitation, and ultraviolet rays. Thus, there is much difference in the internal structural distribution and aging between the lab-fabricated asphalt mixture specimens and those in the field. Consequently, many researchers have shifted focus to the asphalt field cores, studying the fracture properties of the cores drilled from the in-service pavement over various durations. 

There are many lab tests to study the fatigue properties of the asphalt mixture, such as the indirect tensile (IDT) test, direct tensile (DT) test, overlay test (OT), four-point bending test, and semi-circular bending (SCB) test. Barman et al. conducted the IDT test to characterize the fatigue resistance of the asphalt mixture and proposed a simple data analysis approach [6]. Luo et al. used the controlled-strain repeated direct tensile test to evaluate the fatigue cracking [7]. Gu et al. conducted the overlay test to investigate the fracture properties of the field-aged asphalt concrete and found that the cracking resistance of the field reduced from 1st month to the 9th month [8]. Kim et al. performed a four-point bending test to investigate the fatigue life of a total of ten asphalt mixtures, including hot-mix asphalt (HMA) and warm-mix asphalt (WMA) with different amounts of reclaimed asphalt pavement (RAP) and recycled asphalt shingles (RAS) [9]. Du et al. performed the SCB test on the layer core samples drilled from five expressways to analyze the sensitivity of fracture energy to factors such as the equivalent single axle load, air void, service age, etc. [10]. These studies demonstrate that the field core samples can reflect the asphalt pavement conditions, and the test results of core samples can be effectively used for the decision-making related to pavement maintenance actions.

Based on the results of the fatigue test of the asphalt mixture, the fatigue cracking models of the asphalt mixtures were developed. The fracture mechanics and the dissipated energy approach are most widely used to evaluate the fatigue resistance of the asphalt mixtures [11]. However, the fatigue test results cannot describe the crack propagation of asphalt mixture at the mesoscopic level. Moreover, it is hard to validate the fatigue cracking models with only a limited number of core samples. 

In recent years, researchers have attempted to use computer technology to simulate the fracture evolution of the asphalt mixture and investigate the various influencing factors on the fatigue behavior. The discrete element method (DEM) has been widely used in pavement engineering since discrete elements can reflect the discontinuous and non-uniform structural characteristics of asphalt mixtures. It can also help reveal the internal structural deformation, cracking, and other mechanical behaviors of asphalt mixtures. Ma et al. built a virtual specimen based on the DEM to estimate the fatigue life of an asphalt mixture and investigated the influence of air void on fatigue life [12]. Xue et al. developed a new approach combining algorithmic techniques and DEM to perform a heterogeneous fracture simulation, and the study proved that the DEM could provide a valid understanding of the fracture behavior of materials so as to be used to diminish the need for numerous laboratory tests [13]. Peng et al. adopted Python language and DEM to generate irregular particles and establish a three-dimensional (3D) discrete element model of asphalt surface to study the mechanical response under different working conditions [14]. However, due to the limitation of obtaining the raw material parameters, most of the existing studies depended on lab-fabricated specimens and were limited to discrete element simulation of the fractures of the core samples. Moreover, it is unclear whether the calibration of the mesoscopic parameters for the simulation through the lab tests can be effectively applied to the limited number of core samples.

The objective of this study is to propose a reliable method to evaluate the fatigue behavior of the asphalt field cores based on discrete element modeling and to conduct mesoscopic contact parameter calibration through lab tests, including the uniaxial compression dynamic modulus test, SCB test, and SCB fatigue test, which can enhance the utilization efficiency of core samples and provide a reliable representation of the fatigue behavior of the core samples. 

This paper is organized as follows. The following section presents the test samples and lab tests conducted to determine the mesoscopic contact parameters in the discrete element modeling. The next section describes the establishment of the virtual specimen using CT scanning of the asphalt field cores and image processing technologies, as well as mesoscopic contact parameter calibration and virtual fatigue tests using discrete element modeling. The fatigue life obtained from the lab test and virtual fatigue test is compared, and the virtual test results of force chains, crack evolution, and displacement are discussed in the following section. The final section summarizes the findings of this study.

## 2. Laboratory Test

The field cores were drilled from the in-service asphalt pavement and used to conduct laboratory tests, including the uniaxial compression dynamic modulus test, SCB test, and SCB fatigue test, to evaluate the material properties of the field cores. The lab test results can be used to calibrate the mesoscopic parameters required in the discrete element modeling. 

### 2.1. Asphalt Field Core

The asphalt field cores used in this study were taken from the Hubei sections of the G4 Beijing–Hong Kong–Macao Expressway, which opened to traffic in 2002. The Hubei sections have an asphalt surface course with a thickness of 16 cm in total. Even though the Hubei sections have been in service for over 20 years, pavement rehabilitation has been undertaken several times, and the originally designed structure of the asphalt surface course has still been chosen. The asphalt surface course are composed of three asphalt layers, i.e., the asphalt surface layer, asphalt middle layer, and asphalt bottom layer, as detailed in Table 1. The asphalt mixture composition was sourced from the original design documents. In the asphalt surface layer, basalt was used, while limestone was used for the asphalt middle and bottom layers. The modified asphalt binder with anti-stripping additives was used, and asphalt content in the mixtures ranged between 3% and 4%.

**Figure 1:**
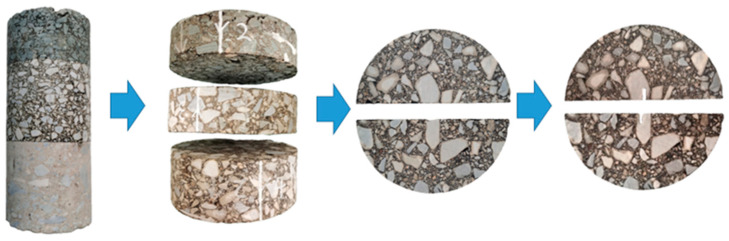
Core sample processing procedure.

The field cores were drilled as cylinders near the wheel path. The cylinder of the field cores is 150 mm in diameter and 30 cm in thickness. The drilled cylinders were first cut into slices 50 mm in thickness and then cut into semi-cylinders as the SCB test specimen in the laboratory. Figure 1 shows the process of the drilled field cores into the SCB test specimen.

### 2.2. Uniaxial Compression Dynamic Modulus Tests

**Figure 2:**
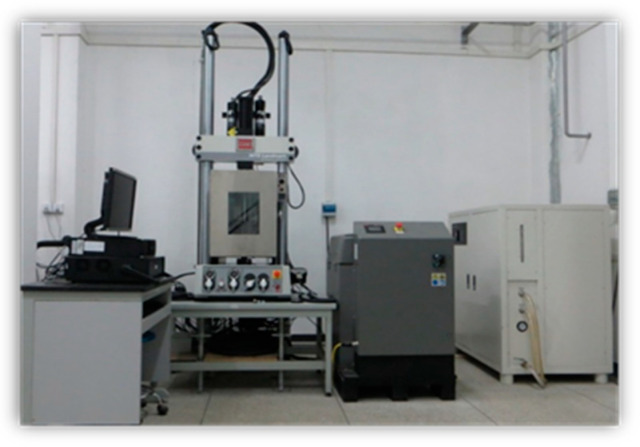
Multifunctional test system.

The uniaxial compression dynamic modulus test was conducted by the multifunctional test system (MTS), as shown in Figure 2. This test was conducted by following the Chinese specification JTG E20-2011 at the temperatures of 5 °C, 20 °C, 35 °C, and 50 °C and frequencies of 0.1 Hz, 0.5 Hz, 1 Hz, 5 Hz, 10 Hz, and 25 Hz [15]. From low to high temperature and from high to low frequency, the sinusoidal load was applied to the specimen under the condition of no side limit, and the dynamic modulus and phase angle of each structural layer of the asphalt mixture were calculated according to the obtained stress–strain data and hysteresis time to quantify the linear viscoelastic mechanical properties of the asphalt mixture. The test results can be used to obtain the parameters of the viscoelastic contact model (e.g., Burgers model) in discrete element simulation. 

### 2.3. SCB Test

**Figure 3:**
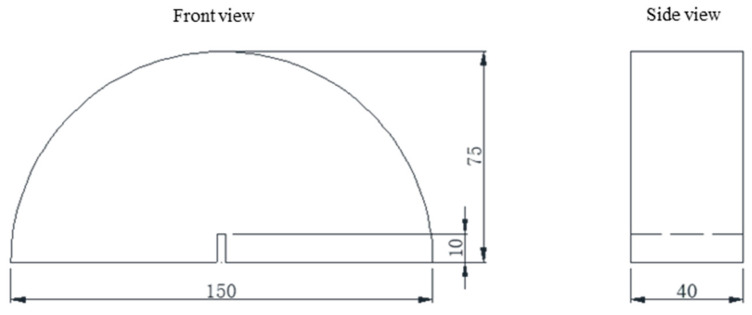
Size of SCB specimen (unit: mm).

The semi-circular bending (SCB) test was conducted by the MTS machine and followed the control mode of the constant displacement rate loading according to the method of TP105-13 [16]. The test was conducted at a constant loading rate of 2 mm/min and a temperature of 20 °C. The dimensions of the semi-circular specimen are 150 mm in diameter, 75 mm in height, and 40 mm in thickness, which align with the thickness of the asphalt surface layer (4 cm). The notch was placed 10 mm in length and 1 mm in width at the center of the specimen to ensure the occurrence of crack initiation and propagation at the notch tip during the test. The test specimens were positioned on two rollers with a distance of 120 mm, which is 0.8 times the diameter of the specimen. Figure 3 shows the dimension of the semi-circular specimen. The SCB test results were obtained to determine the parameters of the parallel bonding model in the discrete element modeling. 

### 2.4. SCB Fatigue Test

The SCB fatigue test was conducted in the loading mode of the stress control with a temperature of 20 °C. The loading mode of the semi-sinusoidal wave at the frequency of 10 Hz was adopted. Considering the cracking self-healing properties of the asphalt mixture, continuous loading without the intermittent time was selected. Since the stress ratio can greatly affect the fatigue life of the asphalt mixture, this study selected the stress ratio of 0.4, 0.5, and 0.6 based on the pre-test results to ensure that fatigue life could fall into the range between thousands and tens of thousands of cycles.

## 3. Discrete Element Modeling

### 3.1. The Establishment of Mesoscopic Virtual Specimen

**Figure 4:**
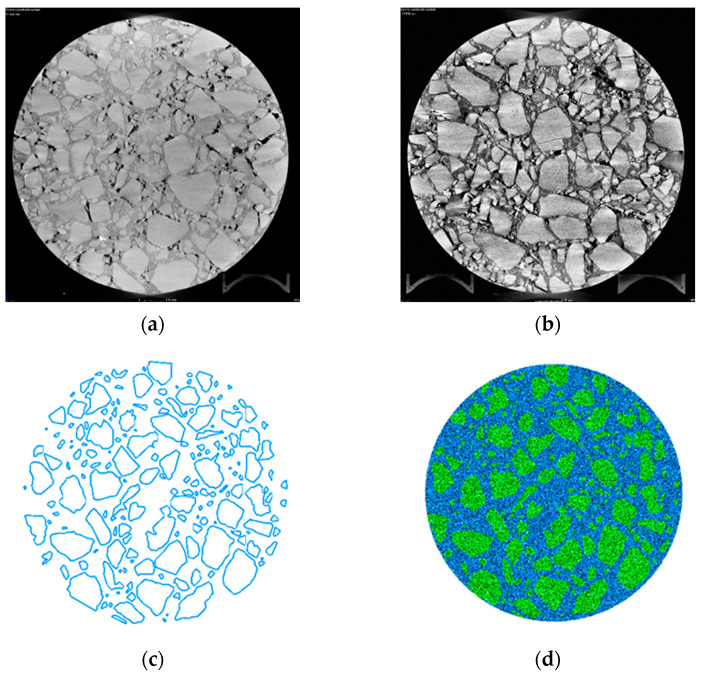
Image processing process. (**a**) Original image. The grayscale image shows the original CT scan of the asphalt core sample. (**b**) The enhanced image. (**c**) Image edge recognition. The blue lines delineate the boundaries of coarse aggregates. (**d**) Complete geometric model. The green area represents the aggregates and blue area represents the asphalt mortar.

The virtual specimen was established by CT scanning and the Digital Image Process (DIP) method. The CT scanning of the asphalt field cores was first conducted, and the scanned image of the field cores was processed by using Image-Pro Plus software (version 7.0). It was found that the air void content of the field cores is higher at both ends compared to the middle, ranging between 3% and 5%. Hence, the median value of 4% was set as the air void content of the virtual specimen for discrete element modeling. The CT-scanned images were proportionally cut by using Photoshop to ensure the geometry of the virtual specimen corresponded to that of the actual specimen. The distribution of aggregate and mortar in the actual specimen was further obtained by using the DIP method, which includes image enhancement, image noise reduction, threshold segmentation, feature extraction, and object recognition. The edge extraction was used to identify the edges of coarse aggregate [17]. The extracted outlines were converted into DXF file format, as shown in Figure 4. 

**Figure 5:**
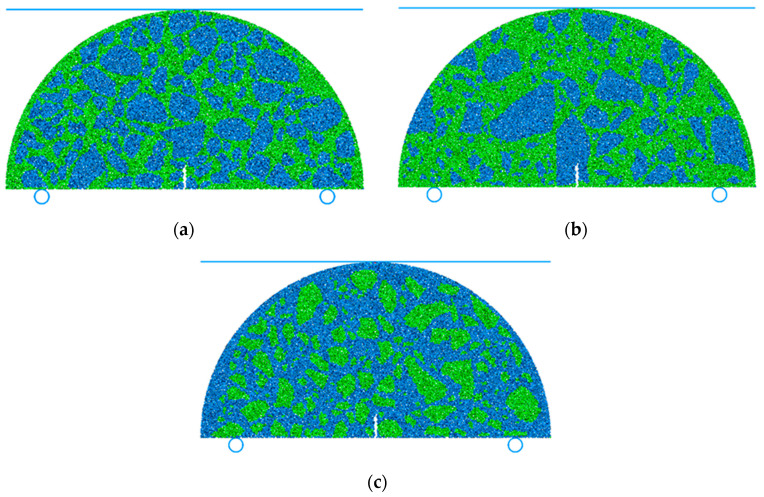
Virtual specimen for virtual SCB fatigue test (**a**) at the asphalt surface layer, (**b**) at the asphalt middle layer, and (**c**) at the asphalt bottom layer. The blue circles at the bottom represent the circular rigid walls as constraints. The blue line at the top represents the wall as a loading plate.

Based on the CT scanning, the images at the center of the asphalt field cores with distinct structures of asphalt layers were selected and then processed. The distribution of coarse aggregates in the field cores was obtained using Photoshop software (version 21.2.11). To simulate the prefabricated notch, the particles at the center bottom of the virtual specimen were deleted. The wall was added at the top of the virtual specimen as a loading plate, and circular rigid walls were added at the bottom as constraints. The loading mode applied on the virtual specimen was consistent with that used in the lab test. The virtual specimens for the asphalt surface, middle, and bottom layers are shown in Figure 5.

### 3.2. Contact Models and Parameter Calibration

The discrete element modeling was performed using the PFC (Particle Flow Code) software (version 5.0). The PFC software has four basic contact models, including the stiffness model, slipping model, parallel bonding model, and viscoelastic contact model. To simplify the contact between the asphalt mixture, the different contact models between the particle elements were selected, as shown in Table 2.

**Figure 6:**
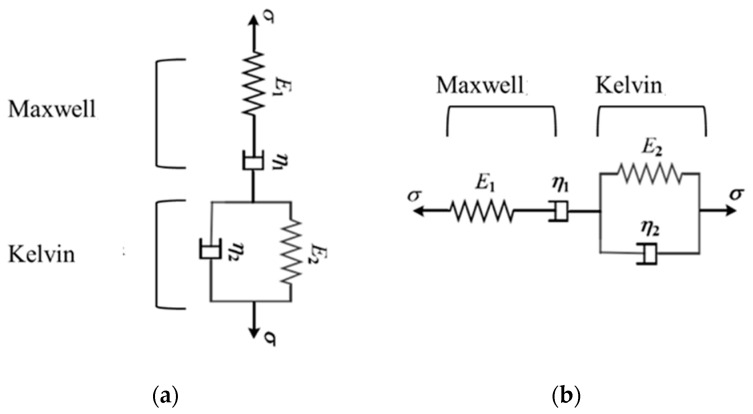
Schematic diagram of Burgers model. (**a**) Normal. (**b**) Shear.

In this study, the viscoelastic behavior of the asphalt mixture is characterized using the Burgers model, which combines the Maxwell model and the Kelvin model, which act in series but in normal and shear directions, as depicted in Figure 6. The Maxwell and Kelvin models both include a spring with stiffness parameters and a dashpot with viscosity parameters. In the Maxwell model, these components are connected in series, while in the Kelvin model, they are connected in parallel. Hence, the Burgers model is divided into normal and shear directions at the mesoscopic level, resulting in eight parameters of the contact model between aggregate and asphalt mortar. These parameters can be calculated by the following equations.

(1) $$C_{\mathrm{mn}}=\eta_{1}LK_{\mathrm{mn}}=E_{1}LC_{\mathrm{kn}}=\eta_{2}LK_{\mathrm{kn}}=E_{2}L$$

(2) $$K_{\mathrm{ms}}=\frac{E_{1}L}{2\left(1+\nu \right)}C_{\mathrm{ms}}=\frac{\eta_{1}L}{2\left(1+\nu \right)}K_{\mathrm{ks}}=\frac{E_{2}L}{2\left(1+\nu \right)}C_{\mathrm{ks}}=\frac{\eta_{2}L}{2\left(1+\nu \right)}$$

where *K*_mn_ and *C*_mn_ are the stiffness and viscosity parameters of the Maxwell model, and *K*_kn_ and *C*_kn_ are the stiffness and viscosity parameters of the Kelvin model in the normal direction; since Burgers model can sustain tensile stress, *K*_ms_ and *C*_ms_ are the stiffness and viscosity parameters of the Maxwell model, and *K*_ks_ and *C*_ks_ are the stiffness and viscosity parameter of the Kelvin model in the shear direction. These parameters of stiffness and viscosity in the Burgers model determine the creep behavior of the virtual specimen. *L* is the length of the two-contacting discrete particle element for aggregate, i.e., the sum of two contacting particle radii; *E*_1_, *η*_1_, *E*_2_, and *η*_2_ are the macro-parameters of the Burgers model.

The macro-parameters of the Burgers model (*E*_1_, *η*_1_, *E*_2_, *η*_2_) were converted from the uniaxial compression dynamic modulus test results of the field core specimen, as shown in Table 3. The macro-parameters of the Burgers model can be calculated by the following equations:

(3) $$E_{1}={\left[\left|E^{*}\right|\right]}_{\omega =\omega_{max}}$$

(4) $$\eta_{1}={\left[\frac{\left|E^{*}\right|}{\omega }\right]}_{\omega =\omega_{min}}$$

(5) $$\frac{1}{\left|E^{*}\right|}=\sqrt{\frac{1}{E_{1}^{2}}+\frac{1}{\eta_{1}^{2}\omega^{2}}+\frac{1+2\left(E_{2}/E_{1}+\eta_{2}/\eta_{1}\right)}{E_{2}^{2}+\eta_{2}^{2}\omega^{2}}}$$

(6) $$tan\;\varphi =\frac{E_{1}\left[E_{2}^{2}+\eta_{2}\left(\eta_{1}+\eta_{2}\right)\omega^{2}\right]}{\eta_{1}\omega \left(E_{2}^{2}+E_{1}E_{2}+\eta_{2}^{2}\omega^{2}\right)}$$

where $\omega_{max}$ and $\omega_{min}$ are, respectively, the maximum and minimum values of angular frequency in the laboratory test. Using 20 °C as an example, the four macro-parameters of the asphalt surface, middle, and bottom layers of the pavement core sample are shown in Table 4. 

Based on the conversion from the macro-parameters (*E*_1_, *η*_1_, *E*_2_, *η*_2_) to the mesoscopic parameters using Equations (1) and (2), the mesoscopic parameters of the contact models were calculated at 20 °C, as shown in Table 5.

### 3.3. Contact Parameter Calibration of Parallel Bonding Model

The parameters of the parallel bonding model were determined based on the SCB test results of the asphalt field cores and discrete element simulation. Firstly, the initial values and range of the parameters were selected based on the previous studies, as shown in Table 6. The parameters of the parallel bonding model were further determined by the trial-and-error approach based on the effect of parameters on the stress–strain curve in the discrete element simulation. Table 7 presents the contact parameters of the parallel bonding model calibrated and validated through the SCB test.

### 3.4. Virtual Fatigue Test

The virtual fatigue test was performed to simulate the SCB fatigue test with the loading mode consistent with that in the laboratory SCB fatigue test. In the virtual fatigue test, the fatigue behavior of the asphalt mixture is characterized by the deterioration at contacts within the asphalt mortar and between the aggregate and asphalt mortar. Hence, the virtual fatigue test of the asphalt mixture was conducted to investigate the deterioration of the mechanical properties of the parallel bonding model in the discrete element simulation. The micro-mechanical fatigue damage model was determined by the following equation [18,19].

(7) $$\frac{d\bar{D}}{dt}=\beta_{1}{\left(\frac{\sigma }{\sigma_{c}}\right)}^{\alpha_{1}}t^{\beta_{2}{\left(\frac{\sigma }{\sigma_{c}}\right)}^{\alpha_{2}}}$$

where *D* is the bonding diameter at contacts; *t* is the loading time; $\sigma_{c}$ is the ultimate tensile strength; σ is the tensile stress between particle elements; and $\beta_{1}$, $\beta_{2}$, $\alpha_{1}$, and $\alpha_{2}$ are the coefficients of the fatigue damage model. The coefficients were initialized based on the existing studies [18,19] and then calibrated through the iterative virtual fatigue test to correspond with the SCB fatigue test results. Table 8 presents the calibrated coefficients of the fatigue damage model.

## 4. Results and Discussion

### 4.1. Comparison between Laboratory and Virtual Fatigue Test

Table 9 presents the comparison of the results of fatigue life between the laboratory test and discrete element modeling at different stress ratios of 0.4, 0.5, and 0.6 for each asphalt structural layer. It was found that fatigue life decreases with the increase in the stress ratio and the depth of asphalt layers. The results from both lab and virtual tests indicate that the fatigue life of the asphalt surface layer was roughly twice as long as that of the asphalt bottom layer. The comparison, as shown in Table 9, also shows that the simulated fatigue life was shorter than the fatigue life from the lab test, which may result from the self-healing properties of the asphalt mortar. The variation in distribution and air void content may contribute to the differences in fatigue life between laboratory and virtual fatigue tests. However, the trend of the result of the virtual fatigue test was consistent with that of the lab test, and the error was below 20%. Compared to the benchmark established in the literature [12], the error range is considered acceptable. The comparison result indicates the virtual fatigue test simulated by discrete element modeling is reliable and acceptable. 

### 4.2. Force Chain Evolution Process

**Figure 7:**
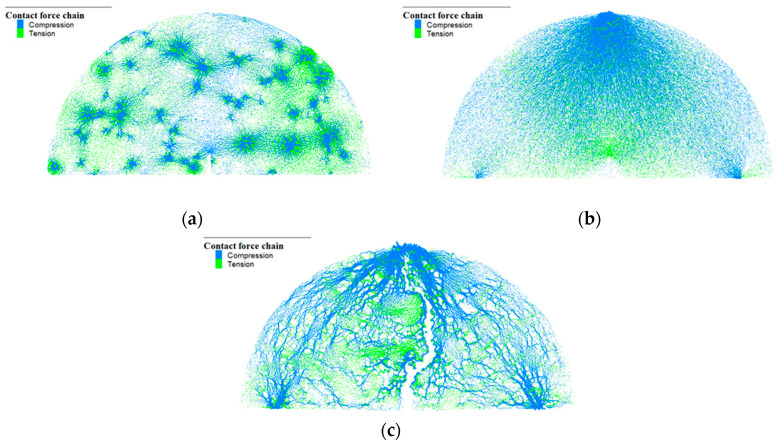
Evolution process of force chain: (**a**) early stage; (**b**) middle stage; (**c**) final stage.

The force chain, formed by the interaction between contacting particles, was analyzed to study the stress distribution of the virtual specimen since the evolution of the force chain reflects the variation in the mechanical response of the virtual specimen during loading. The process of force chain at three stages, i.e., the early, middle, and final stages (i.e., the crack initiation, crack propagation, and failure), is illustrated in Figure 7. The blue and green denote compressive and tensile stress, respectively, and the line size of the force chain reflects the stress level between particle contacts. It is observed from Figure 7 that in the early stage, the compressive force chains are primarily located near the aggregate particles. In the middle stage, the compressive force chains were concentrated to the loading point at the top and two bottom supports. The tensile force chains mainly appear near the notch cracks at the bottom center of the virtual specimen. In the final stage, the virtual specimen eventually failed due to fracture damage. It was found that crack propagation is mainly caused by concentrated tensile stress during loading. 

Table 10 presents the distribution of the force chain under different stress ratios. We utilized the “Contact force chain” command in the PFC (Particle Flow Code) software to record the force chain evolution under loading. The term “proportion of the tensile force chain” in Table 10 refers to the percentage of the tensile force chains to all force chains. The variation in the proportion of the tensile/compressive force chains can reflect the mechanical response of the virtual specimen under loading, which can help to evaluate the fatigue behavior of the virtual specimen. It is observed that with the increase in the stress ratio, the tensile and compressive force chains increase slightly, and the proportion of the tensile force chains rises from 37.4% to 40.41%. The peak values of the force chains were observed as the tensile force chain of 5.18 × 10^4^, 6.62 × 10^4^, and 7.54 × 10^4^ at the stress ratio of 0.4, 0.5, and 0.6, respectively. This indicates that crack propagation is closely associated with the growth of the tensile chains, which suggests that the crack propagation stage could consume a significant portion of the fatigue life since tensile force mainly increases in this stage.

### 4.3. Crack Evolution Process

**Figure 8:**
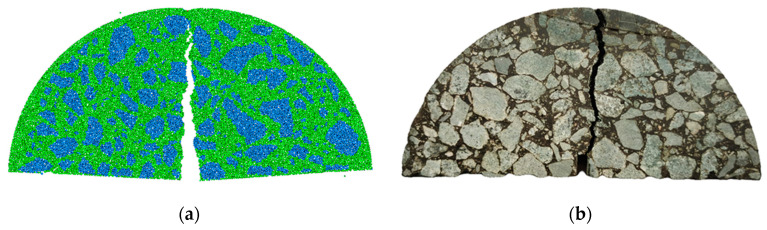
Crack propagation path: (**a**) virtual path. (**b**) test path.

Figure 8 shows the crack path of both virtual and lab specimens. It was found that the two crack paths are relatively consistent, and both of them first appear at the prefabricated notch and then gradually propagate to the top along the middle of the specimen. In the virtual test, the crack path turned close to a straight line, and the internal crack of the aggregate interface and asphalt mortar interface happened simultaneously. By contrast, in the lab test, the crack path seemed to wiggle and mostly grew along the aggregate interface. 

**Figure 9:**
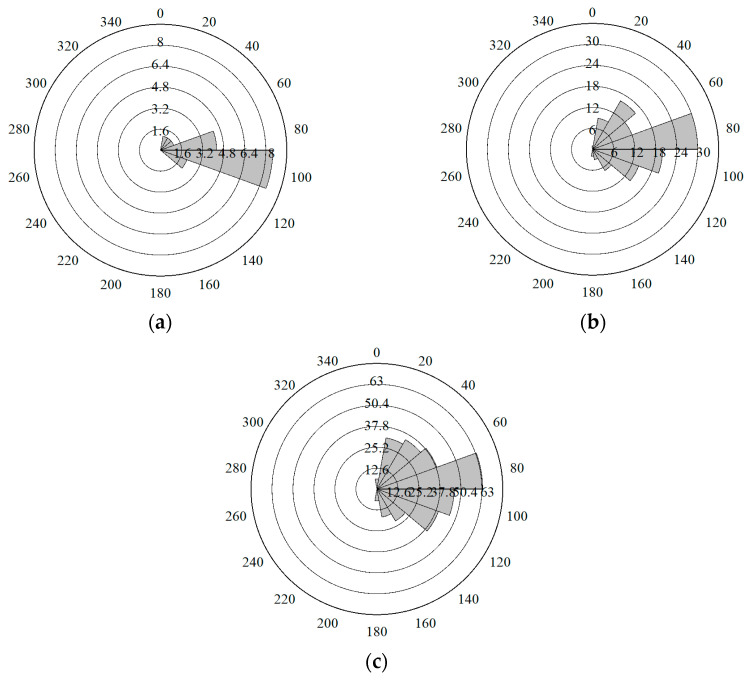
Crack quantities and directions in (**a**) early stage, (**b**) middle stage, and (**c**) final stage.

Figure 9 shows the crack quantities and direction at three stages (i.e., the early stage, middle stage, and final stage) under loading. In Figure 9, it can be seen that in the early stage, cracks mainly grow vertically, and the direction of crack growth ranges between 90 and 110 degrees. At this stage, the cracks have not yet propagated upward at the prefabricated notch. In the middle stage, the crack growth deviates from the vertical direction and ranges from 60 to 100 degrees. In the final stage, the crack gradually propagates to the top of the specimen, and the direction of crack growth extends to a range between 50 and 130 degrees. The extension of the crack growth angle results in the formation of micro-crack branches. 

### 4.4. Displacement Evolution Process

**Figure 10:**
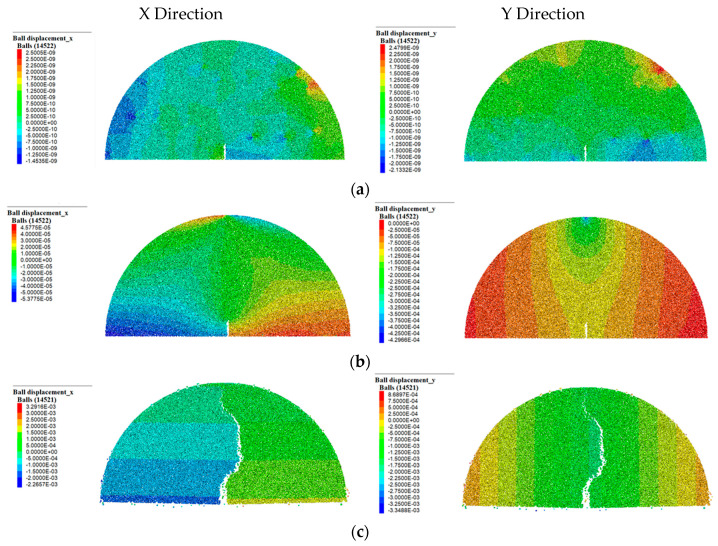
Displacement field of virtual specimen in (**a**) early stage, (**b**) middle stage, and (**c**) final stage.

The displacement field in the discrete element modeling is crucial to studying the movement of particle elements under loading and further evaluating fatigue behavior. Figure 10 shows the displacement field in the early, middle, and final stages of loading. It was found that the horizontal and vertical displacement fields of particles seem asymmetrical and close to zero, which indicates that the relative motion between particles is small. In the middle stage, the displacement between particles is symmetrically distributed with the central axis of the virtual specimen. In the horizontal direction (X direction), the relative displacement at the bottom near the prefabricated notch is the largest, about 0.25 mm. In the vertical direction (Y direction), the displacement at the loading head seems largest, about 0.43 mm. In the final stage, the displacement near the notch reaches about 2 mm in the horizontal direction. In the vertical direction, the displacement near the crack path appears consistent, yet the displacement field seems tortuous along the symmetry axis of the central axis due to the variation of the air void distribution. The vertical displacement near the loading head is about 2.2 mm, which shows a good agreement with the results of the SCB fatigue test. 

## 5. Conclusions

In this study, the virtual SCB fatigue test was simulated by using discrete element modeling to evaluate the fatigue behavior of the asphalt field cores. The CT scan test was conducted to build the mesoscopic geometric model of the asphalt field cores. Additionally, the uniaxial compression dynamic modulus test and SCB test were performed to determine the parameters of the contact model in the virtual fatigue test. Based on the virtual SCB fatigue test, the displacement and contact forces, as well as crack growth, were analyzed. The main findings of this study can be drawn as follows.

(1) The evaluation methodology of fatigue behavior of the asphalt field cores based on the discrete element simulation was developed and can be used to enhance the effective usage of the field cores, which can help with the decision-making of pavement maintenance actions.
(2) The fatigue life simulated in the virtual fatigue test was consistent with that of the laboratory SCB fatigue test. The error between the simulated and test fatigue life was below 20%, which shows that the virtual fatigue test result is acceptable and reliable.
(3) It was found from the analysis of the force chain evolution process that concentrated tensile stress during loading can lead to crack initiation and propagation, ultimately resulting in material failure.
(4) The fatigue cracks in the asphalt mixture were observed as the three stages, i.e., crack initiation, crack propagation, and failure. It was found that the crack propagation stage consumes a significant portion of the fatigue life since tensile contact force mainly increases in this stage.

In this study, the discrete element modeling was restricted within 2D simulation due to the limited computational power. In future work, the 3D discrete element simulation will be performed to evaluate the fatigue behavior of the asphalt field cores, which could further improve the simulation accuracy. Additionally, future work will compare the asphalt field cores with the different aging times and investigate the difference in the fatigue behavior among them.

## Figures and Tables

**Table 1:** Structure of the asphalt surface course.

No.	Directions	Materials	Thickness (cm)
1	Asphalt surface layer	SUP-12.5	4
2	Asphalt middle layer	AC-20I	6
3	Asphalt bottom layer	AC-20S	6

**Table 2:** Selection of contact model between particle elements.

Particle Elements	Contact Model Selection
Between coarse aggregate particle units	Linear stiffness model
Between coarse aggregate and asphalt mortar	Burgers model + parallel connection model
Between asphalt mortar	Burgers model + parallel connection model
Between the particle unit and the wall	Linear stiffness model

**Table 3:** Dynamic modulus test results of each structural layer of the core sample at 20 °C.

Frequency	Asphalt Surface Layer		Asphalt Middle Layer		Asphalt Bottom Layer	
	Dynamic Modulus, MPa	Phase Angle	Dynamic Modulus, MPa	Phase Angle	Dynamic Modulus, MPa	Phase Angle
25 Hz	16,174	12.58	12,128	15.33	12,669	12.87
10 Hz	13,199	13.51	10,478	16.89	11,551	14.45
5 Hz	11,503	14.72	9152	18.52	10,172	15.64
1 Hz	8214	19.83	6199	24.15	7030	20.41
0.5 Hz	6871	21.90	5119	26.72	5855	22.60
0.1 Hz	4567	23.23	3177	30.66	3844	25.58

**Table 4:** Macroscopic parameters of the contact Burgers model at 20 °C.

Model Parameter	Asphalt Surface Layer	Asphalt Middle Layer	Asphalt Bottom Layer
$E_{1}$/GPa	14.96	12.46	12.88
$E_{2}$/GPa	10.72	7.08	8.98
$\eta_{1}$/(GPa·s)	23.89	11.93	17.70
$\eta_{2}$/(GPa·s)	1.22	0.82	0.99

**Table 5:** Calibration results of meso-contact parameters in Burgers model at 20 °C.

Meso-Structure Parameter	Asphalt Surface Layer	Asphalt Middle Layer	Asphalt Bottom Layer
*K*_mn_	1.70 × 10^7^	1.25 × 10^7^	1.50 × 10^7^
*C*_mn_	2.10 × 10^7^	1.10 × 10^7^	1.50 × 10^7^
*K*_kn_	9.00 × 10^6^	8.00 × 10^6^	1.20 × 10^7^
*C*_kn_	1.45 × 10^6^	9.50 × 10^5^	1.10 × 10^6^
*K*_ms_	6.00 × 10^6^	5.00 × 10^6^	5.20 × 10^6^
*C*_ms_	1.00 × 10^7^	1.20 × 10^7^	7.00 × 10^6^
*K*_ks_	4.20 × 10^6^	2.80 × 10^6^	3.60 × 10^6^
*C*_ks_	5.00 × 10^5^	3.50 × 10^5^	4.00 × 10^5^

**Table 6:** Initial values of main parameters of the parallel bonding model.

Parameter Label	Parameter	Unit	Initial Value	Value Range
pb_emod	Parallel bond modulus	Pa	6.00 × 10^5^	6.00 × 10^4^~8.00 × 10^6^
pb_ten	Strength of extension	Pa	8.00 × 10^5^	8.00 × 10^4^~8.00 × 10^6^
pb_coh	Bonding force	Pa	4.00 × 10^5^	4.00 × 10^4^~4.00 × 10^6^
pb_krat	Stiffness ratio	/	2.0	1~3
pb_fa	Internal friction angle	°	35	–
pb_rad	Parallel bond radius	mm	0.5	–

**Table 7:** Contact parameter values of parallel bonding model.

Parameter Label	Asphalt Surface Layer	Asphalt Middle Layer	Asphalt Bottom Layer
pb_emod	3.20 × 10^5^	7.40 × 10^5^	3.20 × 10^6^
pb_ten	8.40 × 10^5^	2.20 × 10^6^	3.80 × 10^6^
pb_coh	1.00 × 10^6^	7.50 × 10^5^	6.50 × 10^5^
pb_krat	2.0	1.7	1.5
pb_fa	35	35	35
pb_rad	0.5	0.5	0.5

**Table 8:** Coefficients of the fatigue damage model.

Structural Layer	$\alpha_{1}$	$\alpha_{2}$	$\beta_{1}$	$\beta_{2}$
Asphalt surface layer	−1.420	0.053	2.20 × 10^6^	−1.066
Asphalt middle layer	−1.350	0.050	2.50 × 10^6^	−1.710
Asphalt bottom layer	−1.435	0.042	3.10 × 10^6^	−1.790

**Table 9:** Comparison of fatigue life between lab and virtual fatigue test.

Asphalt Structure Layer	Stress Ratio	Test Fatigue Life	Simulated Fatigue Life	Error (%)
Asphalt surface layer	0.6	4310	3587	16.77
	0.5	6867	5388	21.54
	0.4	24,478	19,566	18.47
Middle layer	0.6	2155	1816	15.73
	0.5	4458	3653	18.06
	0.4	12,135	9585	21.01
Bottom layer	0.6	1751	1950	11.36
	0.5	4274	3653	17.00
	0.4	10,097	8086	19.91

**Table 10:** Distribution of force chains under different stress ratios.

Stress Ratio	Tensile Force Chain	Tensile Force Chain Proportion (%)	Compressive Force Chain	Compressive Force Chain Proportion (%)
0.4	11,843	37.40	19,819	62.60
0.5	13,536	40.22	20,121	59.78
0.6	13,688	40.41	20,185	59.59

## Data Availability

Data are contained within the article.
